# Cardiovascular Autonomic Responses to Aerobic, Resistance and Combined Exercises in Resistance Hypertensive Patients

**DOI:** 10.1155/2022/8202610

**Published:** 2022-04-20

**Authors:** Nayara Fraccari-Pires, Hélio José Coelho-Júnior, Bruno Bavaresco Gambassi, Ana Paula Cabral de Faria, Alessandra Mileni Versuti Ritter, Carolina Souza Gasparetti, Mariana Rodrigues Pioli, Olívia Moraes Ruberti, Silvia Elaine Ferreira-Melo, Heitor Moreno, Bruno Rodrigues

**Affiliations:** ^1^Laboratory of Cardiovascular Pharmacology, School of Medical Sciences, University of Campinas (UNICAMP), Campinas, Brazil; ^2^Università Cattolica Del Sacro Cuore, Rome, Italy; ^3^Laboratory of Cardiovascular Investigation & Exercise (LICE), School of Physical Education (FEF), University of Campinas (UNICAMP), Campinas, Brazil

## Abstract

Here, we report the acute effects of aerobic (AER), resistance (RES), and combined (COM) exercises on blood pressure, central blood pressure and augmentation index, hemodynamic parameters, and autonomic modulation of resistant (RH) and nonresistant hypertensive (NON-RH) subjects. Twenty participants (10 RH and 10 NON-RH) performed three exercise sessions (i.e., AER, RES, and COM) and a control session. Hemodynamic (Finometer®, Beatscope), office blood pressure (BP), and autonomic variables (accessed through spectral analysis of the pulse-to-pulse BP signal, in the time and frequency domain-Fast Fourrier Transform) were assessed before (T0), one-hour (T1), and twenty-four (T2) hours after each experimental session. There were no changes in office BP, pulse wave behavior, and hemodynamic parameters after (T0 and T1) exercise sessions. However, AER and COM exercises significantly reduced sympathetic modulation in RH patients. It is worth mentioning that more significant changes in sympathetic modulation were observed after AER as compared to COM exercise. These findings suggest that office blood pressure, arterial stiffness, and hemodynamic parameters returned to baseline levels in the first hour and remained stable in the 24 hours after the all-exercise sessions. Notably, our findings bring new light to the effects of exercise on RH, indicating that RH patients show different autonomic responses to exercise compared to NON-RH patients. This trial is registered with trial registration number NCT02987452.

## 1. Introduction

Hypertension is one of the most prevalent clinical conditions in adults [1]. Approximately 20% of the hypertensive patients do not achieve adequate blood pressure (BP) levels, regardless of the optimal dose of 3 or more antihypertensive drugs, including one diuretic, denoting resistant hypertension (RH). These patients have been associated with negative-health-related outcomes and increased risk of death [2].

In this context, researchers have been looking for alternative therapies besides pharmacological treatment to manage BP in RH and nonresistant hypertensive (NON-RH) patients. Physical exercise has been highlighted among the many available alternatives due to evidence supporting the beneficial effects of exercise training programs on BP in NON-RH [3]. The beneficial effects of physical exercise are not restricted to chronic programs, and low BP levels are also observed after an acute session of exercise, which is called postexercise hypotension [4–10].

According to MacDonald [11], postexercise hypotension may allow a better understanding of mechanisms and implications of regulation of BP after exercise. Additionally, autonomic modulations exert an essential role in the physiopathology of NON-RH and RH [12, 13], and improvements in autonomic modulation are a well-established mechanism associated with postexercise hypotension in normotensive and NON-RH subjects [14].

In addition, it should be stressed that the acute effects of physical exercise are associated with chronic adaptations [9, 15]. In this sense, in an elegant study performed by Eches et al. [15], the acute reduction of systolic BP after exercise may be considered a reliable predictor of the chronic adaptations to training in older women.

In fact, previous studies have demonstrated effects of different exercise training programs on the BP and autonomic modulation of normotensive and/or prehypertensive individuals [16–19]. Although cardiovascular effects have been found after exercise in NON-RH, these findings may differ in RH patients due to different pathological bases.

In this sense, Santos et al. [20] found significant postexercise hypotension in RH patients who performed low- and moderate-intensity aerobic exercise. Additionally, evidences have demonstrated that aerobic training has a strong capacity to collaborate with changes on BP in RH patients [21, 22]. Although some studies have investigated the impacts of aerobic exercise, the effects of resistance exercises on BP and autonomic modulation in this population remain relatively unexplored in the literature.

In view of the health challenges affecting the RH patients, the recommendation of the American College of Sports Medicine (ACSM) [23] [practice of aerobic exercises (3 to 5 days per week) associated with resistance exercises (2 to 3 days per week)], and the scarcity of studies using resistance exercises in this population, we decided to carry out the present research. Thus, the primary outcome of this study was to evaluate the blood pressure and autonomic responses after aerobic, resistant, and combined exercises in resistant and nonresistant hypertensives. We also demonstrated the pulse wave behavior after exercise sessions in these patients.

## 2. Methods

### 2.1. Sample and Recruitment

This is a randomized crossover trial that determined the effects of an acute session of aerobic (AER), resistance (RES), and combined (COM) exercises on cardiovascular responses of RH and NON-RH people.

Using a convenience sampling, we recruited patients of the Outpatient RH Clinic of the University of Campinas (Campinas, SP, Brazil). Twenty patients agreed to participate in the study protocol: 10 nonresistant hypertensive patients (NON-RH), in medical follow-up at the Outpatient RH Clinic for at least one year; and 10 were clinically diagnosed with RH. RH was defined as an uncontrolled BP despite using ≥3 antihypertensive medications at optimal doses, including a diuretic if possible, or patients with controlled BP using ≥4 antihypertensive medications [2]. The diagnosis of RH was assessed following a 6-month protocol for screening of secondary causes of hypertension (primary hyperaldosteronism, renal artery stenosis, pheochromocytoma, Conn's or Cushing's syndrome, and obstructive sleep apnea) and pseudo-RH (counting pills and ambulatory blood pressure monitoring (ABPM)). Participants carrying one or both conditions were properly excluded from the study. Patients who showed significant changes on electrocardiogram trace (mainly on ST-segment, suggesting myocardial ischemia) under resting or during the physical stress test, antihypertensive medication in the past 6 months, cardiac or cerebrovascular diseases, heart failure or renal dysfunction, practicing regular physical exercise over the 6 months preceding the beginning of the study, using hormonal replacement therapy, and smokers were also excluded. We included male and female patients aged from 40 to 80 years old able to practice physical exercises.

This study was approved by the Research Ethics Committee of the Faculty of Medical Sciences, University of Campinas (Campinas, Brazil) (Protocol 1638486; registered at ClinicalTrials.gov under ID number NCT02987452). The investigation was performed according to the Helsinki Declaration of 1975 (as revised in 1983).

### 2.2. Procedures

Experiments were performed in two distinct phases in a quiet, air-conditioned room (22-24°C) always in the mornings (07:00-12:00 am). In the first phase, participants were familiarized with the physical exercises used in the present study, and the optimal loads to aerobic (AER), resistant (RES), and combined (COM) exercises were determined. This period occurred over 2 weeks. The second phase was composed of 5 visits. Participants arrived in the laboratory after a 12-hour overnight fast, including water, and were advised to avoid energetic and alcohol consumption and intense physical activity for 24 hours before the experimental session.

Anthropometric parameters and blood collection (for biochemical analyses) were assessed in the first visit. Anthropometric parameters were measured using a Bioimpedance Analyzer 450 (Biodynamics Corporation, Seattle, USA) and included body mass index (BMI), fat-free mass, fat mass, basal metabolic rate, and total body water content.

The second visit served to baseline (T0) evaluations, which were performed in the following order: (1) office BP, (2) applanation tonometry analyses with Beatscope system, (3) hemodynamic parameters, and (4) autonomic modulation assessment.

The third, fourth, and fifth visits to the laboratory occurred on nonconsecutive days to perform the different types of exercises. At least 1 hour after a standardized light breakfast (i.e., 25 g chocolate minicookie [Bauducco, São Paulo, Brazil], 200 mL chocolate box milk [Toddynho, PepsiCo, São Paulo, Brazil], and 26 g brown crackers pack [Club Social Nabisco, São Paulo, Brazil]), participants performed an acute session of exercise (i.e., AER, RES, or COM) according to prior randomization. Office BP, hemodynamic parameters, and autonomic modulation were assessed again in the first hour (T1) and 24 hours (T2) after exercise, while applanation tonometry was only reanalyzed at T1.

### 2.3. Office Blood Pressure

Office BP was measured using a certified digital sphygmomanometer (HEM-907 XL OMRON Healthcare Inc., Bannockburn, IL, USA) by a trained health professional, according to the European Society Hypertension (ESH) 2018 guidelines [24]. After remaining seated on a comfortable recliner chair for 15 min in a quiet room, an appropriate cuff was placed in the arm about 2 cm from the antecubital fossa. Measurements of BP were performed on both arms with a time difference of 1 minute between procedures. Additionally, new measurements were performed more twice in the arm with the highest BP values. The mean of these BP values was used to represent office BP.

### 2.4. Pulse Wave Analyses (PWAs) and Central Blood Pressure

Applanation tonometry was performed to assess noninvasive central hemodynamic variables and pulse wave analyses (PWAs) using the SphygmoCor system (AtCor Medical, Sydney, Australia). Consecutive measurements of the carotid pulse waves were electrocardiogram gated. After 20 sequential waveforms were acquired and averaged, a validated generalized mathematical transfer function was used to synthesize the corresponding central aortic pressure wave [25].

The augmentation index (AIx), defined by the ratio between the pressure exerted by the reflected wave and the ejection wave, was evaluated [25].

### 2.5. Hemodynamic and Autonomic Modulation

With patients in a sitting position, after 15 minutes of rest, continuous beat-to-beat blood pressure waves were obtained by a digital photoplethysmography device (Finometer®, Finapress Medical System BV, Netherlands) for 20 minutes. A software program (BeatScope) used BP curves and patient data (age, sex, body mass, and stature) to calculate systolic and diastolic BP (SBP and DBP), heart rate (HR), cardiac output (CO), and peripheral vascular resistance (PVR). The waveforms were simultaneously recorded on another computer equipped to acquire and convert the biological signals AT/MCA-CODAS (DATAC Instruments Inc., Akron, Ohio, USA). The sampling frequency of signals was 1000 Hz.

The stored data from photoplethysmography underwent a routine analysis (spectral analysis) to provide pulse interval (PI) and systolic blood pressure (SBP) variability. Although the PI variability assessment may be considered less accurate than measuring heart rate variability by electrocardiogram, some studies have demonstrated the agreement between heart rate variability and PI variability [26, 27].

Beat-to-beat BP was analyzed using a specialized algorithm for MATLAB MT (MATLAB 6.0, Mathworks, USA), which automatically detects SBP and DBP waves. Pulse interval (PI) was calculated as the difference between the cycle's start and endpoints (T1-T0). The spectral power density of the SBP and the PI range were computed using the Fast Fourier Transform and the Welch method. Setting the window length was established in 5 minutes, excluding the first and last 7.5 minutes.

In the time domain, we analyzed the following: SDNN (standard deviation of normal-to-normal (NN) PI) and VAR PI (total variance of PI); RMSSD (the square root of the mean of the sum of the squares of differences between adjacent NN intervals, which represents cardiac vagal modulation of PI); and VAR SAP (variance of systolic blood pressure in short-time). The spectral bands evaluated for humans were defined as very-low-frequency (VLF: 0.007–0.04 Hz), low-frequency (LF: 0.04–0.15 Hz), high-frequency (HF: 0.15–0.4 Hz), and total power. The normalized values (nu) for the LF and HF bands were then calculated using the predefined formulae: LF (n.u.) = LF/(total power spectral density − VLF) × 100 or HF (n.u.) = HF/(total power spectral density − VLF) × 100. The ratio for the absolute values for the LF band of PI and HF band of PI (LF/HF) was also calculated as a representative of autonomic balance. Spontaneous baroreflex sensitivity was assessed through the alpha index (R-R LF ms^2^/LF mmHg^2^) [28].

The HF component of PI variability has been related to the efferent vagal modulation. However, the interpretation of the LF component of PI is more controversial since that includes the influences of sympathetic and parasympathetic modulation [29]. Also, there is evidence that the LF component of SBP variability is influenced by sympathetic modulation of vascular tone and myogenic vascular function [30]. Furthermore, the assessment of blood pressure variability in very short-term (beat-to-beat) reflects the influences of central and reflex autonomic modulation, elastic properties of arteries, and humoral and emotional factors [31].

### 2.6. Laboratory Assessments

Blood samples were collected by venipuncture in heparinized vacutainers after 12 h fasting and immediately centrifuged at 4000 rpm for 5 min to separate plasma. Plasma aldosterone concentration was measured by radioimmunoassay (Immunotech SAS, Marseille, France) according to the manufacturer's instructions. Creatinine clearance (mL/min/1.73 m^2^) was measured in urine sample collected during 24 h.

### 2.7. Exercise Protocols

Exercise protocols were based on the American College of Sports and Medicine (ACSM) guidelines [32, 33]. The different types of exercise (i.e., AER, RES, and COM) were equalized according to the total session time. A minimum interval of 96 hours was required between the sessions. AER session was performed in an electronic treadmill (Life Fitness®, model 9700HR®, Fort Mill, Tennessee, USA) for 45 minutes at 50-60% of maximal HR (HRmax) obtained from the ergometric stress test. HR was continuously monitored throughout the exercise session using a cardiac monitor (Polar RS800 CX, Polar Electro Oy, Kempele, Finland).

RES consisted of 6 exercises with 4 sets of 12 submaximal repetitions at moderate intensity (3-5 on the adapted Borg scale) (Foster et al., 2001). Exercises were performed in the following order: (1) chair squat, (2) vertical bench press, (3) seated knee raise, (4) seated row, (5) dorsiflexion and plantar flexion, and (6) shoulder abduction. A 1 min rest interval was adopted between sets and exercises. All exercises were performed in the full range of motion, and muscle contractions—concentric and eccentric—were performed at moderate velocity (2 sec for each). Participants were instructed to avoid the Valsalva maneuver during the full muscle contraction, regardless of the exercise session.

COM consisted of AER exercise performed at 50-60% HRmax for 25 minutes plus RES based on 6 exercises with 2 sets of 12 submaximal repetitions at moderate intensity according to modified Borg scale [34]. All exercise sessions lasted up to 60 minutes and were supervised by an exercise physiologist.

Optimal training load for RES and COM sessions was acquired during the familiarization period using the rating of perceived exertion (RPE) method [35] based on the resistance of the elastic bands proposed and recommended by [36–38]. A maximal exercise stress test using an individualized incremental protocol on a treadmill was performed to determine AER and COM intensities. Electrocardiogram, BP, HR, and lactate levels were assessed at rest after a 20 min rest. The incremental test was based on the modified Bruce protocol, which includes six stages with 3 min each, characterized by increasing speed (2.7-6.8 km/h) and grade (0-16%). The HRmax was considered the highest HR recorded at the exhaustion moment. Electrocardiograph patterns were registered and accompanied by a cardiologist throughout the whole test.

### 2.8. Control Session (CONT)

In the CONT, participants remained seated in the machines without exercising for approximately 60 min.

### 2.9. Statistical Analysis

The normality of data was tested using the *Shapiro-Wilk* test. Baseline comparisons between RH and NON-RH were performed using unpaired Student's *t*-test. A three-way ANOVA followed by a Bonferroni post hoc test was performed to identify differences among the different times of evaluations in the groups. Categorical variables were presented in frequencies and/or percentages and compared by the chi-square test. Cohen's effect size (ES) *d* was calculated to assess the magnitude of the results according to the following formula: $\text{T}0–\text{T}1\text{ or T}2/\left(\sqrt{\left[\left(\text{SD}12+\text{SD}22\right)/2\right]}\right)$.

The level of significance was set at 5% (*P* < 0.05), and all statistical analyses were performed using GraphPad Prisma 6.0 (GraphPad Prism Inc., 2000).

The sample size was estimated using G∗Power version 3.1.9.2 based on the magnitude of the mean differences in SBP scores among the three sessions in two repeated measures. Considering an ES set at 0.45, a power of 80%, and a level of significance set at 5%, the sample size was estimated to be 10 participants. These estimates were based on SBP changes in response to AER reported by [20], given that no prior studies investigated the acute effects of AER, RES, and COM in RH and NON-RH.

## 3. Results

The general characteristics of RH and NON-RH participants are shown in Table 1. Clinical and biochemical parameters were similar between RH and NON-RH. However, NON-RH tends to show higher glucose levels than RH, while RH tends to show higher HDL-c levels. As expected, all RH patients were under diuretic treatment. However, diuretics were only taken by 5 NON-RH patients, and it was lower than in RH (*P* = 0.03). No other differences were found among the groups. There were no dropouts in the study and any patient-reported changes on antihypertensive medication during the follow-up examination.

**Figure 1:**
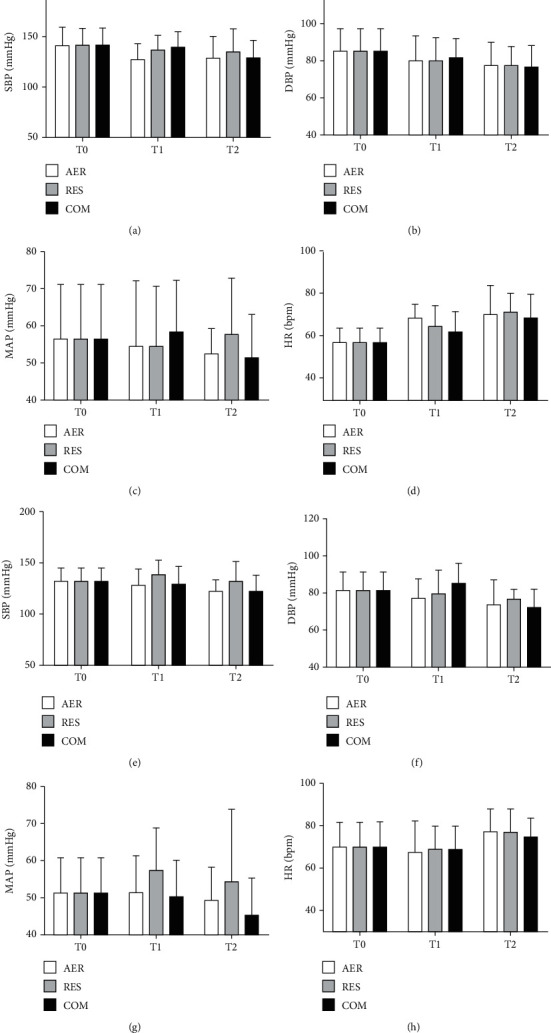
Effects of exercise sessions on office blood pressure in RH (a–d) and NON-RH (e–h) at baseline (T0), postexercise (T1), and 24 hs (T2) after exercise sessions. AER: Aerobic; RES: resistance; COM: combined; SBP: systolic blood pressure; DBP: diastolic blood pressure; MAP: mean arterial pressure; HR: heart rate.

**Figure 2:**
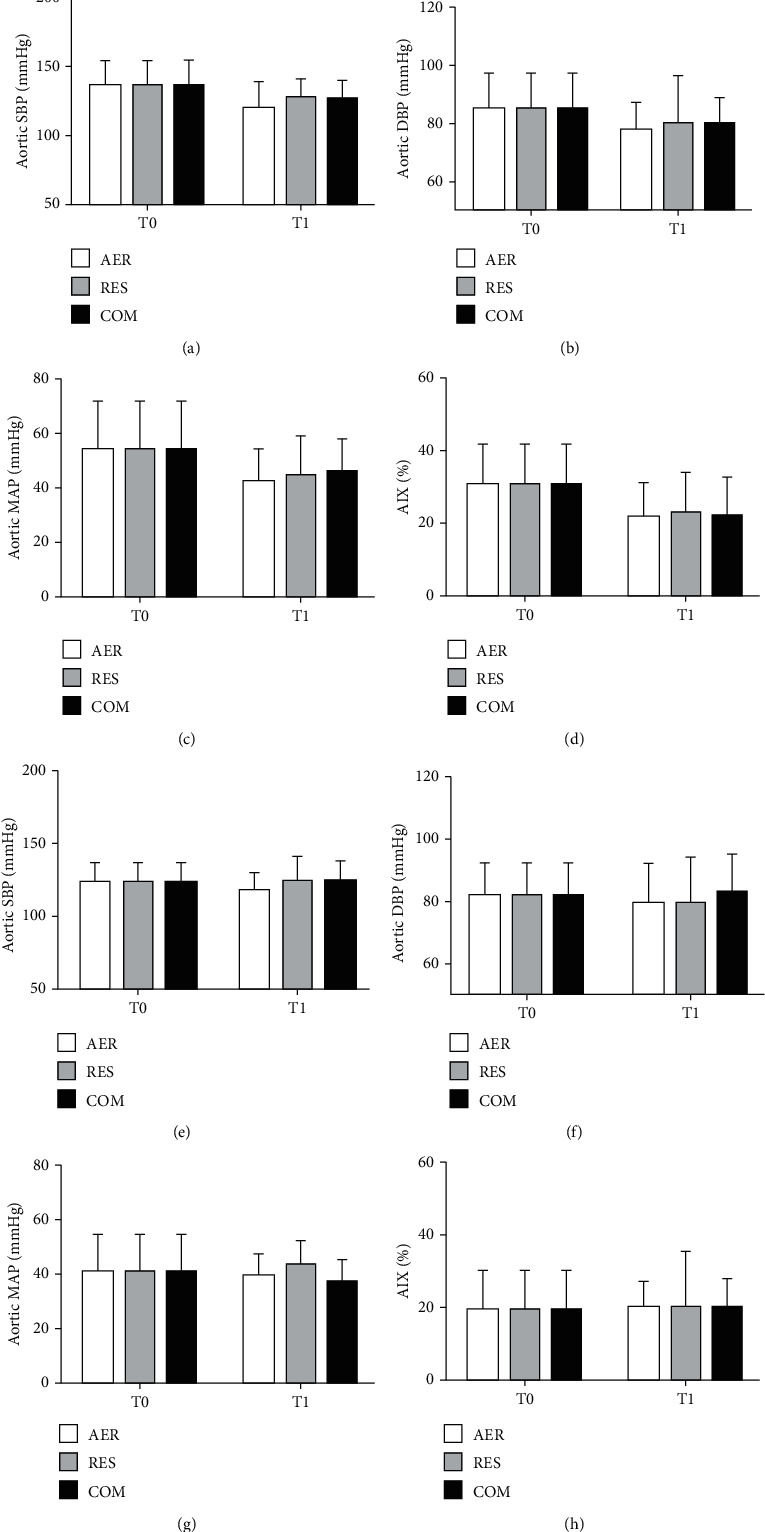
Effects of exercise sessions on central blood pressure and augmentation index in RH (a–d) and NON-RH (e–h) at baseline (T0) and postexercise (T1). AER: aerobic; RES: resistance; COM: combined; SBP: systolic blood pressure; DBP: diastolic blood pressure; MAP: mean arterial pressure; AIx: augmentation index.

**Figure 3:**
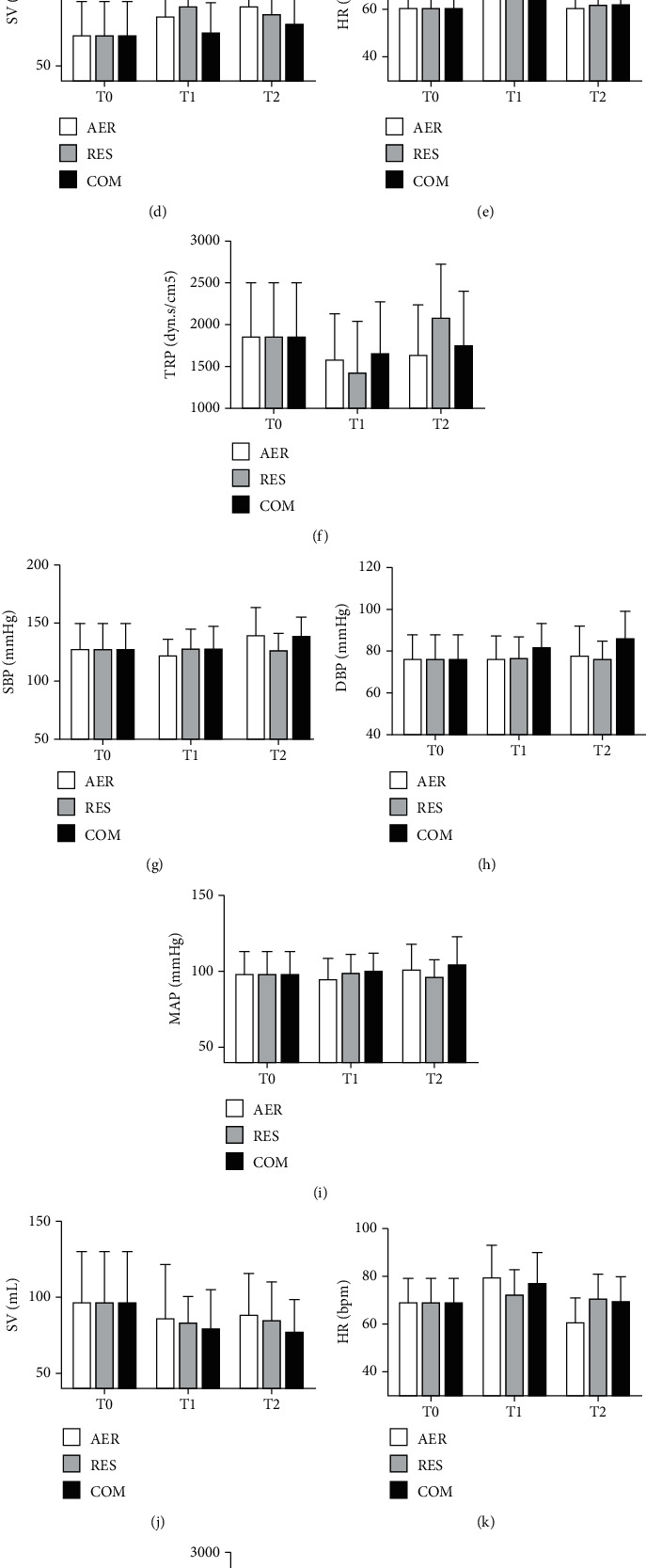
Effects of exercise sessions on hemodynamic parameters in RH (a–g) and NON-RH (h–n) at baseline (T0), postexercise (T1), and 24 hs (T2) after exercise sessions. AER: aerobic; RES: resistance; COM: combined; SBP: systolic blood pressure; DBP: diastolic blood pressure; MAP: mean arterial pressure; SV: stroke volume; CO: cardiac output; HR: heart rate; TRP: total peripheral vascular resistance.

The effects of AER, RES and COM exercises on office blood pressure, central blood pressure, augmentation index, and hemodynamic parameters in RH and NON-RH are shown in Figures 1–3, respectively. At T0, NON-RH showed plethysmography values and office heart rate and lowered augmentation index when compared to RH. No further between- and within-group differences were observed.

Effect size results for RH and NON-RH are shown in Tables 2 and 3, respectively. In RH, nonsignificant changes on systolic, diastolic, and mean blood pressures evaluated by both plethysmography and oscillometric methods. However, central systolic and diastolic blood pressures had higher effect size classification immediately (T1) after AER session in comparison to RES and COM, while COM showed a higher effect size classification in heart rate comparing AER and COM exercises. Except for a large effect size on heart rate (T1 evaluation) in the AER session and in systolic blood pressure and pulse pressure after COM, similar effect size classifications were observed among exercise sessions in NON-RH.

Tables 4 and 5 show pulse interval- and systolic blood pressure-variability in RH and NON-RH, respectively. Lower high frequency (ms^2^) was found in NON-RH in comparison to RH at baseline. Variance of pulse interval was significantly reduced at T1 after AER and COM in RH. However, a higher variance o pulse interval was observed in COM when compared to AER at T2. AER also caused reductions in low-frequency band (in % and ms^2^), and high-frequency (ms^2^) at T2 in RH. In addition, the root mean square of the successive differences was significantly reduced at T1 after AER in NON-RH. No other between- and within-group differences were observed.

## 4. Discussion

The main findings of the present study indicate that cardiac workload, assessed through office BP, PWAs, and hemodynamic parameters, returned to baseline (T0) levels in the first hour (T1) and remained stable in the 24 hours after all exercise sessions, independently of hypertension status. Nevertheless, different patterns of autonomic modulation were observed among the groups. Indeed, sympathetic modulation to the heart was significantly reduced after AER and COM in RH, with a more significant effect been observed after AER. On the other hand, AER caused a slight but significant decrease in vagal modulation (RMSSD) in the first hour after the exercise session in NON-RH.

SBP and DBP at T1 and T2 were similar to baseline levels in all exercise groups, suggesting the absence of postexercise hypotension. This interpretation is supported by the lack of changes in hemodynamic parameters (i.e., CO, PVR, and SV) after exercise. By contrast, many investigations have demonstrated postaerobic [5, 39], postresistance [7, 40] and postcombined [41, 42] exercise hypotension in NON-RH, and a recent seminal study found significant postaerobic exercise hypotension in RH patients [20].

Dissimilarities may explain differences between prior studies and the present study in gender [5, 43], baseline blood pressure levels [7, 44], pharmacological therapy [45], times of assessment [5], and design of the exercise program [5, 18, 19, 46]. To note, NON-RH and RH were under rigorous pharmacological therapy, which results in their well-controlled BP and may prevent PEH. In addition, the time of cardiovascular assessment seems to be crucial to identify PEH, given that some researchers [15, 40] suggested that postexercise hypotension last for approximately 60 minutes or less, and our evaluations were performed in the first hour and 24 hours after the exercise session.

Nevertheless, the cardiac workload may remain elevated for more than one hour after the exercise session [47]. This phenomenon is not attractive, mainly in populations with a high cardiovascular risk, such as NON-RH and RH. Therefore, although findings of the present study refute the hypothesis that an acute session of AER, RES, and COM exercises can elicit postexercise hypotension in NON-RH and RH, our exercise sessions seem to be safe by patients with high cardiovascular risk, so that their chronic effects should be investigated for better conclusions.

Our results indicate that AER and COM caused different autonomic responses in RH and NON-RH, suggesting that the severity of hypertension may influence the cardiovascular responses to exercise. Although sympathetic overactivity exerts a significant role in the physiopathology of NON-RH and RH, its bases seem to be different among the conditions. In RH, an autonomic imbalance in favor of sympathetic overactivity is commonly caused in response to hyperaldosteronism [12, 13] while many factors—from genetics to environmental—have been mentioned in the context of essential hypertension [48].

Unfortunately, the possible mechanisms responsible for these different autonomic responses were not investigated in the present study, limiting our inferences. Nevertheless, AER caused more significant autonomic effects in comparison to COM in RH. Interestingly, earlier investigations found lower postexercise hypotension after COM when compared to AER in normotensive and hypertensive patients [41, 42], and researchers suggested that postaerobic exercise hypotension may be blunted in COM due to the RES component. Indeed, RES might cause cardiac overactivity over one hour after the end of the exercise session. On the other hand, the reduced sympathetic activity to the heart observed at T1 seems important and may collaborate to postexercise hypotension through a significant reduction in HR and SV, consequently reducing CO. However, other mechanisms probably counterbalanced the changes in autonomic modulation elicited by AER preventing postexercise hypotension.

The present study has some limitations that should be acknowledged, such as the absence of more assessment times, the inclusion of obese individuals, and the lack of assessment of other possible mechanisms associated with postexercise hypotension. Notably, AER seems to cause more significant nonsignificant reductions in SBP, DBP, MAP, and aortic SBP and DBP compared to RES and COM. However, these findings are not supporting by the hypothesis test, indicating that more studies with larger sample sizes are still necessary.

## 5. Conclusion

Findings of the present study suggest that office blood pressure, central blood pressure, and hemodynamic parameters returned to baseline levels in the first hour and remained stable in the 24 hours after the all-exercise sessions. In addition, our results bring new light to the effects of an acute session of exercise on hypertension, indicating that different autonomic responses to exercise are observed between RH and NON-RH patients.

## Figures and Tables

**Table 1:** General characteristics of resistant hypertensive subjects (RH) and nonresistant hypertensive subjects (NON-RH).

	RH (*n* = 10)	NON-RH (*n* = 10)	*P* value
*Clinical data*			
Age (years)	60 ± 9	54 ± 13	0.66
Female gender, *n* (%)	6 (60)	5 (50)	1
Diabetes mellitus, *n* (%)	10 (100)	5 (50)	0.03
BMI (kg/m^2^)	31 ± 5	32 ± 7	0.18
Free-fat mass (kg)	54 ± 18.5	61 ± 10.6	0.40
Fat mass (kg)	25 ± 10	26 ± 14	0.32
Total body water (L)	75 ± 2	75 ± 3	0.17
Basal metabolic rate (cals/day)	1765 ± 482	1996 ± 540	0.39
Office SBP (mmHg)	147 ± 11	134 ± 8	0.02
Office DBP (mmHg)	85 ± 7	78 ± 6	0.08
Office MBP (mmHg)	56 ± 22	49 ± 10	0.12
Office HR (bpm)	59 ± 6	70 ± 9	0.03
*Biochemical data*			
HbA1C (%)	7 ± 2	6 ± 0.7	0.13
Glucose (mg/mL)	97 ± 25	98 ± 20	0.07
Creatinine (mg/mL)	0.8 ± 0.2	0.8 ± 0.2	0.81
Aldosterone (pg/mL)	100 ± 141	132 ± 95	0.64
Creat Clear (mL/min/1.73m^2^)	83 ± 66	89 ± 65	0.86
Cholesterol (mg/mL)	188 ± 48	175 ± 40	0.55
HDL-c (mg/mL)	44 ± 9	38 ± 7	0.07
LDL-c (mg/mL)	109 ± 35	106 ± 46	0.88
Triglycerides (mg/mL)	125 ± 75	143 ± 108	0.97
*Anti-HT drugs*			
Number of classes	4 ± 1	2 ± 1	0.13
Diuretics, *n* (%)	10 (100)	5 (50)	0.03
Spironolactone, *n* (%)	2 (20)	2 (20)	1
Beta-blockers, *n* (%)	8 (80)	6 (60)	0.63
ACEIs and ARBs, *n* (%)	5 (50)	7 (70)	0.65
CCBs, *n* (%)	8 (80)	3 (30)	0.07
Others, *n* (%)	0	0	1

According to data distribution, values are expressed as mean ± standard deviation or median (1st, 3rd quartiles). RH: resistant hypertensive subjects; NON-RH: nonresistant hypertensive subjects; BMI: body mass index; SBP: systolic blood pressure; DBP: diastolic blood pressure; MBP: mean blood pressure; HR: heart rate; HbA1C: glycated hemoglobin; Creat Clear: creatinine clearance; LDL and HDL: low- and high-density lipoproteins, respectively; antiHT: antihypertensive drugs; ACEIs: angiotensin-converting enzyme inhibitors; ARBS: angiotensin receptor blockers; CCBs: calcium channel blockers.

**Table 2:** Effect size for hemodynamic parameters after the experimental sessions in RH.

		AER	RES	COM
*Photoplethysmography*				
SBP	T1	0.87 (large)	0.31 (small)	0.27 (small)
	T2	0.33 (small)	-0.03	0.57 (medium)
DBP	T1	0.51 (medium)	0.65 (medium)	0.25 (small)
	T2	0.32 (small)	-0.17	0.78 (medium)
MAP	T1	0.80 (large)	0.35 (small)	0.46 (small)
	T2	0.40 (small)	-0.09	0.80 (large)
SV	T1	0.06	0.11	0.69 (medium)
	T2	-0.16	0.20 (small)	0.22 (small)
CO	T1	0.33 (small)	0	0.5 (medium)
	T2	0	0.39 (small)	0.5 (medium)
HR	T1	-0.63 (medium)	-0.62 (medium)	-0.66 (medium)
	T2	0		
TPR	T1	0.11	0.55 (medium)	0.52 (medium)
	T2	-0.25 (small)	-0.51 (medium)	0.62 (medium)
Office				
SBP	T1	0.51 (medium)	0.40 (small)	0.35 (small)
	T2	0.66 (medium)	0.43 (small)	0.77 (medium)
DBP	T1	0.65 (medium)	0.43 (small)	0.47 (small)
	24	0.63 (medium)	0.69 (medium)	0.78 (medium)
PP	T1	0.32 (small)	0.34 (small)	0.11
	T2	0.47 (small)	0.17	0.56 (medium)
HR	T1	-0.76 (medium)	-0.75 (medium)	-0.91 (large)
	T2	-1.17 (large)	-1.53 (large)	-1.11 (large)
*Applanation tonometry*				
Central SBP	T1	0.84 (large)	0.59 (medium)	0.59 (medium)
Central DBP	T1	0.65 (medium)	0.31 (small)	0.49 (small)
Central PP	T1	0.78 (medium)	0.60 (medium)	0.52 (medium)
AIx	T1	0.89 (large)	0.72 (medium)	0.81 (large)

AER: aerobic; RES: resistance; COM: combined; SBP: systolic blood pressure; DBP: diastolic blood pressure; MAP: mean arterial pressure; PP: pulse pressure; SV: stroke volume; CO: cardiac output; HR: heart rate; TPR: total peripheral vascular resistance; AIx: augmentation index; T1: postexercise; T2 : 24 hours after exercise protocols.

**Table 3:** Effect size for hemodynamic parameters after the experimental sessions in NON-RH.

		AER	RES	COM
*Photoplethysmography*				
SBP	T1	0.28 (small)	-0.44 (small)	-0.29 (small)
	T2	-0.29 (small)	0.27 (small)	-0.46 (small)
DBP	T1	0.20 (small)	-0.09	-0.34 (small)
	T2	-0.17	0	-0.59 (medium)
MAP	T1	0.26 (small)	0.07	-0.32 (small)
	T2	-0.23 (small)	0.23 (small)	-0.37 (small)
SV	T1	0.20 (small)	0.35 (small)	0.33 (small)
	T2	-0.11	0.24 (small)	0.27 (small)
CO	T1	0	0	-0.5 (medium)
	T2	-0.19	0.5 (medium)	-0.5 (medium)
HR	T1	-0.82 (large)	-0.16	-0.47 (small)
	T2	0.09	0	0.17
TPR	T1	-0.00	0.61 (medium)	-0.32 (small)
	T2	-0.46 (small)	0.58 (medium)	-0.74 (medium)
Office				
SBP	T1	0.46 (small)	-0.12	0.24 (small)
	T2	0.97 (large)	0.31 (small)	0.85 (large)
DBP	T1	0.09	0	-0.09
	T2	0.65 (medium)	0.60 (medium)	0.34 (small)
PP	T1	0.32 (small)	-0.15	0.41 (small)
	T2	0.50 (medium)	0.05	0.82 (large)
HR	T1	0	0.08	-0.30 (small)
	T2	-0.60 (medium)	-0.77 (medium)	-0.47 (small)
*Applanation tonometry*				
Central SBP	T1	0.35 (small)	-0.13	0.13
Central DBP	T1	0.25 (small)	0	-0.09
Central PP	T1	0.18	-0.17	0.35 (small)
AIx	T1	0	0	0

AER: aerobic; RES: resistance; COM: combined; SBP: systolic blood pressure; DBP: diastolic blood pressure; MAP: mean arterial pressure; PP: pulse pressure; SV: stroke volume; CO: cardiac output; HR: heart rate; TPR: total peripheral vascular resistance; AIx: augmentation index; T1: postexercise; T2 : 24 hours after exercise protocols.

**Table 4:** Pulse interval and systolic blood pressure variability parameters of RH at baseline (T0), postexercise (T1), and 24 hs (T2) after exercise sessions.

	Var-IP (ms^2^)	RMSSD (ms)	LF (ms^2^)	LF (%)	HF (ms^2^)	HF (%)	LF/HF	Var-SBP (mmHg^2^)	LF (mmHg^2^)	AI, LF, (ms/mmHg)
AER										
T0	2339 ± 1734	47 ± 32	751 ± 440	58 ± 19	898 ± 1258	41 ± 19	1.9 ± 1.2	73 ± 40	27 ± 8	5 ± 1
T1	982 ± 592^a^	25 ± 16	488 ± 434	59 ± 30	280 ± 360	40 ± 30	3.8 ± 3.6	75 ± 46	45 ± 34	3 ± 1
T2	2034 ± 1320	65 ± 42	367 ± 388^a^	32 ± 17^a^	715 ± 602^a^	67 ± 17	0.5 ± 0.4	62 ± 13	11 ± 9	6 ± 3
RES										
T0	2269 ± 1750	59 ± 60	464 ± 393	45 ± 21	651 ± 604	54 ± 21	1.2 ± 1.3	83 ± 54	9 ± 6	8 ± 4
T1	1991 ± 2656	33 ± 18	833 ± 1549	58 ± 21	422 ± 546	41 ± 21	1.8 ± 1.2	89 ± 53	9 ± 7	6 ± 3
T2	2880 ± 2381	52 ± 34	546 ± 424	43 ± 21	882 ± 1035	56 ± 21	1.1 ± 1.0	69 ± 22	12 ± 9	6 ± 3
COM										
T0	3342 ± 1535	56 ± 28	961 ± 789	44 ± 17	1393 ± 985	55 ± 17	0.9 ± 0.5	75 ± 27	16 ± 4	7 ± 3
T1	1513 ± 939^a^	31 ± 17^a^	446 ± 600	45 ± 25	620 ± 966	54 ± 25	1.5 ± 1.8	79 ± 21	19 ± 5	7 ± 13
T2	3843 ± 2459^b^	43 ± 22	838 ± 692	47 ± 15	905 ± 844	52 ± 15	1.0 ± 0.5	87 ± 19	12 ± 7	10 ± 9

AER: aerobic; RES: resistance; COM: combined; Var-IP: variance of pulse interval; RMSSD: root mean square of the successive differences; LF: low-frequency band; HF: high-frequency band; Var-SBP: variance of systolic blood pressure; AI: alpha index; T0: baseline; T1: immediately after; T2: 24 hs after. ^a^*P* < 0.05 vs. T0; ^b^*P* < 0.05 vs. AER.

**Table 5:** Pulse interval and systolic blood pressure variability parameters of NON-RH at baseline (T0), postexercise (T1), and 24 hs (T2) after exercise sessions.

	Var-IP (ms^2^)	RMSSD (ms)	LF (ms^2^)	LF (%)	HF (ms^2^)	HF (%)	LF/HF	Var-SBP (mmHg^2^)	LF (mmHg^2^)	AI, LF, (ms/mmHg)
AER										
T0	3919 ± 2705	47 ± 22	608 ± 526	43 ± 14	911 ± 833	56 ± 14	0.9 ± 0.7	50 ± 23	22 ± 12	5 ± 1
T1	2227 ± 2038	22 ± 9^a^	812 ± 1459	55 ± 15	741 ± 1358	44 ± 15	1.6 ± 1.2	44 ± 19	24 ± 10	3 ± 2
T2	2639 ± 1793	34 ± 14	436 ± 376	44 ± 17	649 ± 519	55 ± 17	1.0 ± 0.8	35 ± 20	18 ± 10	4 ± 1
RES										
T0	2786 ± 2183	34 ± 15	438 ± 374	52 ± 16	434 ± 436	47 ± 16	1.4 ± 1.2	56 ± 27	29 ± 17	3 ± 1
T1	1963 ± 1484	28 ± 12	361 ± 288	52 ± 14	370 ± 341	47 ± 14	1.4 ± 1.1	47 ± 23	25 ± 11	4 ± 2
T2	2261 ± 1702	31 ± 15	329 ± 282	50 ± 17	477 ± 482	49 ± 17	1.2 ± 1.1	40 ± 21	20 ± 9	4 ± 2
COM										
T0	2776 ± 1416	45 ± 18	740 ± 564	57 ± 12	496 ± 392^b^	42 ± 12	1.2 ± 0.4	67 ± 34	12 ± 8	7 ± 4
T1	1561 ± 1105	35 ± 25	301 ± 271	55 ± 17	294 ± 300	44 ± 17	1.6 ± 1.4	58 ± 33	12 ± 9	5 ± 3
T2	2696 ± 1716	36 ± 11	506 ± 429	53 ± 14	489 ± 435	46 ± 14	1.6 ± 1.5	80 ± 69	12 ± 10	6 ± 3

AER: aerobic; RES: resistance; COM: combined; Var-IP: variance of pulse interval; RMSSD: root mean square of the successive differences; LF: low-frequency band; HF: high-frequency band; Var-SBP: variance of systolic blood pressure; AI: alpha index; T0: baseline; T1: immediately after; T2: 24 hs after. ^a^*P* < 0.05 vs. T0; ^b^*P* < 0.05 vs. AER.

## Data Availability

Data are available on request.
